# Study on urinary metabolomics of premenstrual dysphoric disorder patients with liver-qi depression syndrome treated with Xiaoyaosan: Study Protocol Clinical Trial (SPIRIT Compliant)

**DOI:** 10.1097/MD.0000000000019425

**Published:** 2020-04-17

**Authors:** Mengbai Xu, Yanfeng Liu, Yu Guo, Chenyue Liu, Yueyun Liu, Zhiyi Yan, Yajing Hou, Xiaojuan Li, Qingyu Ma, Xuan Zhou, Liuqing Liu, Sheng Huang, Jiaxu Chen

**Affiliations:** a School of Traditional Chinese Medicine, Beijing University of Chinese Medicine; b Dongzhimen Hospital Affiliated to Beijing University of Chinese Medicine, Beijing; c School of Traditional Chinese Medicine, Jinan University, Guangzhou; d Jiuzhitang Co., Ltd., Changsha, China.

**Keywords:** herb medicine, premenstrual dysphoric disorder, prospective pilot trial, traditional Chinese medicine, urinary metabolomics, Xiaoyaosan

## Abstract

**Introduction:**

Premenstrual dysphoric disorder (PMDD) is a serious form of premenstrual syndrome with mental symptoms as its main manifestation, which seriously affects women's health and daily life. Some basic research and clinical studies have shown that the Chinese herbal medicine of Xiaoyaosan can relieve the symptoms of mental disorders with few side effects. The aim of this study is to evaluate the clinical efficacy of Xiaoyaosan for treating PMDD with liver-qi depression syndrome. In addition, metabonomics and small molecular marker compounds closely related to the pathogenesis of PMDD are expected to be found, and mechanism of Xiaoyaosan is further explored from the metabolic level.

**Methods and analysis:**

This study is a clinical pilot trial. Thirty PMDD patients with liver-qi depression syndrome and thirty healthy participants will be recruited. Study participants will be assigned in a 1:1 ratio to 2 groups: a normal control group and Xiaoyaosan treatment group. The treatment group will receive the Chinese patent medicine of Xiaoyaosan for 3 menstrual cycles. The primary outcome is the syndrome change in the Daily Record of Severity of Problems (DRSP). The secondary outcome is improvement in TCM syndrome, which will be measured with TCM symptom score scale. Urine metabolism profiles of participants by liquid chromatograph-mass spectrometer (LC-MS) method will be measured to explore the mechanism of PMDD pathogenesis and action of Xiaoyaosan on PMDD.

**Discussion:**

This trial will evaluate the effectiveness and the therapeutic mechanism from the metabolomics level of Xiaoyaosan in individuals with PMDD. If successful, the outcome of this trial will provide a viable treatment option for PMDD patients and objective evidence on the efficacy of Xiaoyaosan for PMDD.

**Ethics and dissemination:**

The trial has been approved by the Institutional Ethics Committee of Dongzhimen Hospital Affiliated to Beijing University of Chinese Medicine (file number: DZMEC-KY-2019-73). Written informed consent will be obtained from all participants. The results of the study will be published in peer-reviewed journals or communicated via yearly reports to funding bodies.

**Trial registration:**

Chinese Clinical Trial Registry, ChiCTR1900026296.

## 1 Introduction

Premenstrual syndrome (PMS) refers to the periodic physical, mental and behavioral changes of women in the luteal phase.^[1]^ Premenstrual dysphoric disorder (PMDD) is a serious form of PMS.^[2]^ Mental symptoms are the main characteristics of PMDD, such as depression, anxiety, irritability, and so on, appearing in the late luteal phase and ending shortly after menstruation.^[3]^ The incidence of PMDD varies from 3% to 8% for premenopausal women.^[4]^ Cyclically recurring premenstrual symptoms seriously affect women's health and daily life,^[5]^ are even related to suicidal ideation.^[6]^ The pathogenesis of PMDD is still insufficiently clear, studies indicate that many factors can cause PMDD, such as hormonal changes, neurotransmitters, diet, stress, and so on.^[7,8]^ The comorbidities of PMDD with other mental disorders are high, a retrospective study showed that more than 50% of women with PMDD had diagnosis of major depression disorder.^[9]^ The therapeutic goal of PMDD is to relieve premenstrual emotional and physical symptoms.^[10]^ In most cases, pharmacotherapy of antidepressants is the main therapy, such as selective serotonin reuptake inhibitors (SSRIs) and other serotonergic antidepressants.^[11]^ The antidepressant medications have positive antidepressant-like effects, but a range of side effects cannot be ignored, such as fatigue, nausea, decreased libido, sweating, and so on.^[11]^

Traditional Chinese medicine (TCM) has been widely used in the treatment of mental disorders in China. An epidemiological study of PMDD based on TCM theories shows that the liver dysfunction is the major pathogenic feature in clinical syndrome differentiation and the liver-qi depression syndrome is an important subtype.^[12]^ Xiaoyaosan, a Chinese herbal formula, was first described in *Taiping Huimin Heji Jufang* during the Song Dynasty of China (960–1127 AD). It has the effect of dispersing stagnated liver qi for relieving qi stagnation. The formula has been used to treat mental disorders for thousands of years in China.^[13,14,15]^ Xiaoyaosan consists of Radix Bupleuri (root of Bupleurum Chinense DC.), Radix Paeoniae Alba (root of Paeonia lactiflora Pall.), Radix Angelicae Sinensis (root of Angelica sinensis (Oliv.) Diels), Rhizoma Atractylodis (root and rhizome of Atractylodes lancea (Thunb.) DC.), Poria (fungus nucleus of Poria cocos (Schw.) Wolf), Radix Glycyrrhizae (root and rhizome of Glycyrrhiza uralensis Fisch.), Herba Menthae (aboveground portions of Mentha haplocalyx Briq.), and Rhizoma Zingiberis Recens (fresh root and rhizome of Zingiber officinaleRosc.) in a ratio of 5:5:5:5:5:4:1:1.^[16]^ At present, Xiaoyaosan is still widely used in clinical treatment of mental disorders. Modern study has shown that Xiaoyaosan can exert antidepressant-like and anxiolytic-like effects effectively. Our research group has reported a large number of studies on the underlying mechanism of action of Xiaoyaosan. For example, Xiaoyaosan can promote the reduction in oxidative-stress-induced hippocampus neuron apoptosis.^[17]^ Xiaoyaosan reduces stress-induced depression by regulating the tryptophan metabolism, Apelin-APJ system, the brain-derived neurotrophic factor (BDNF) and its receptors, and the expression of normalized glial fibrillary acidic protein in hippocampus.^[18–21]^ In addition, Xiaoyaosan can exert antidepressant-like effects by reducing glutamate-induced neuronal damage of frontal cortex and improving the functions of astrocytes and the astrocytic excitatory amino acid transporters.^[22]^ Xiaoyaosan can also improve depressive-like behavior with the modulation of gut microbiota.^[23]^ Similarly, Xiaoyaosan can downregulate the TNF-α/JAK2-STAT3 pathway in the hippocampus and regulate the corticotropin-releasing factor 1 receptor (CRF1R) signaling pathway to reduce stress-induced anxiety behaviors.^[13,24]^

## 2 Objectives

This study aims to evaluate the clinical effectiveness of Xiaoyaosan for treating PMDD patients with liver-qi depression syndrome. In addition, metabonomics and small molecular marker compounds closely related to the pathogenesis of PMDD are expected to be found, and mechanism of Xiaoyaosan is further explored from the metabolic level. To achieve this aim, a non-randomized controlled, prospective, pilot trial has been planned.

## 3 Methods and analysis

### 3.1 Trial design

**Figure 1:**
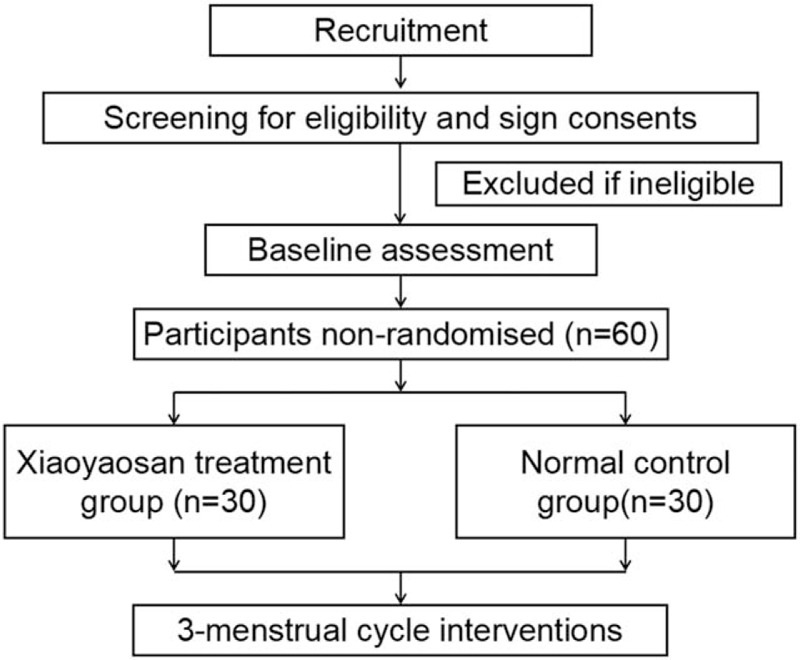
Flow diagram of study design.

This is a non-randomized, controlled, prospective, pilot trial designed to evaluate the effectiveness of Xiaoyaosan in treating PMDD with liver-qi depression syndrome for woman. Thirty PMDD participants with liver-qi depression syndrome and thirty healthy participants will be recruited from Beijing University of Chinese Medicine and Dongzhimen Hospital Affiliated to Beijing University of Chinese Medicine. After obtaining written informed consent from participants, 60 participants will be assigned to Xiaoyaosan treatment group and normal control group, respectively with a ratio of 1:1. The intervention period is 3 menstrual cycles. Figure 1 shows the study flow. The schedule of trial enrolment, interventions and assessments is provided in Table 1. The protocol was designed in accordance with the Consolidated Standards of Reporting Trials guidelines.^[25]^

**Table 1:**
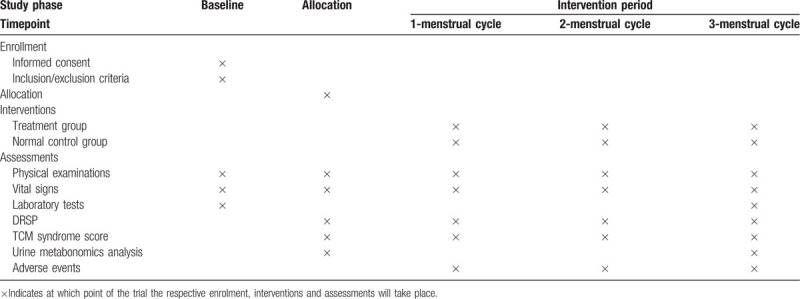
Schedule of enrolment, interventions, and assessments.

### 3.2 Setting and recruitment

The study will be conducted in Beijing University of Chinese Medicine and Dongzhimen Hospital Affiliated to Beijing University of Chinese Medicine. Participants will be recruited through Internet advertisement and posters in the university and hospital. In addition, some participants will be recommended via doctors from the gynecology clinic. Those who are willing to participate in this study can contact the researchers by telephone. Participants will be provided detailed information about the study by the researchers.

## 4 Participants

### 4.1 Diagnostic criteria

The Clinical diagnostic criteria of PMDD is based on the 5th edition of the *Manual of Diagnosis and Statistics of Mental Diseases* (DSM-5).^[2]^ The TCM syndrome of liver-qi depression in the study follows the *Guidelines for Clinical Research of new*

*Chinese Medicine*.^[26]^ In addition, PMS severity criteria should also be met, including the follows:

1. A postmenstrual phase (days 6–10) daily average ≤2.5 for each symptom.
2. A premenstrual phase (day −5 to −1 before menstruation) daily average ≥3.0 for 3 distinct items.
3. A premenstrual phase daily average worsened by at least 50% compared with the corresponding postmenstrual phase daily average for 3 distinct items.
4. A score ≥3 on functional impairment items for at least 1 day during the premenstrual phase.

The diagnostic standards of TCM syndrome of liver-qi depression are as follows:

1. Primary symptoms and signs include premenstrual depression and (or) irritability, breast pain, chest tightness, and ability reduction of work, family management, learning and communicate.
2. Secondary symptoms and signs include chest and hypochondriac distention pain, sighing frequently, lower abdomen falling distention, anorexia, fatigue, menstrual abdomen pain, menstrual blood suspending fluid retention, retarded menstruation.
3. In addition, the patient has a pale or dark tongue, fat body of tongue, thin white or yellow coated tongue, and a stringy thin or stringy slow pulse.

Patients who meet the following criteria can be diagnosed as PMDD liver-qi depression syndrome: 3 primary and secondary symptoms/signs, combined with the tongue and pulse conditions, symptoms appearing before menstruation and disappearing after menstruation for 2 menstrual cycles or more continuously.

### 4.2 Inclusion criteria

Participants of PMDD with liver-qi depression syndrome will be included:

1. Clinical diagnostic criteria of PMDD according to the DSM-5.
2. Women aged between 18 and 30 years.
3. Regular menstrual cycle of 21 to 35 days (fluctuation range <3d).
4. Clear mind and independent judgement.
5. Able to sign informed consent and cooperate voluntarily.

Participants of healthy people will be included:

1. Female college students and graduate students, aged between 18 and 30 years.
2. Regular menstrual cycle (21–35 days).
3. Clear mind and independent judgement.
4. Able to sign informed consent and cooperate voluntarily.
5. Physical health (Consistent with World Health Organization definition of Health).

### 4.3 Exclusion criteria

1. Drugs for PMDD were used within 3 months.
2. History of drug abuse.
3. Those who participated in clinical trials of other drugs within the previous month.
4. Serious hepatic disease, heart disease, kidney disease, blood system disease or malignant disease.
5. Currently pregnant or breast-feeding.
6. Ovariectomy, abortion or contraceptive use within 6 months.

### 4.4 Intervention

Sixty participants will be assigned to Xiaoyaosan treatment group and normal control group respectively with a ratio of 1:1. Participants in Xiaoyaosan treatment group will be required to take Chinese patent medicine with Xiaoyaosan (Xiaoyao Pills, 8 pills) orally with warm water 3 times a day for 3 menstruation cycles continuously. The medicine will be taken from the luteal phase and stopped during the menstrual period of each menstruation. The Xiaoyaosan will be manufactured as pills and provided by Jiuzhitang Co., Ltd. Participants in normal control group will not be intervened. Any other treatments for PMDD will not be allowed during the study. If the condition deteriorates, participants can withdraw from the study and choose modern drug therapy, such as SSRI antidepressants. Study visits will take place at baseline and at 1, 2, 3 menstrual cycle.

## 5 Outcome measure

### 5.1 Primary outcome

The primary outcome measure is the 21 symptom items of the Daily Record of Severity of Problems (DRSP).^[27]^ The symptoms of 60 participants will be evaluated at baseline and at 1, 2, 3 menstrual cycle by the DRSP. The average of the late luteal (premenstrual) phase for symptom items will be summed to generate the total score. The primary efficacy variable is the changes in symptom score between baseline and after 3 menstrual cycles of Xiaoyaosan treatment. In addition, the symptom score of Xiaoyaosan treatment group will also be compared with the normal control group.

### 5.2 Secondary outcome

The secondary outcome is the improvement of TCM syndrome in TCM symptom score scale. The PMDD syndrome scores of TCM will be recorded by the same researcher before and after 3 menstrual cycles treatment to assess whether the treatment improved the patient's signs of PMDD. The evaluation of TCM syndrome improvement will be measured by the reduction (reduction = (before treatment score − after treatment score) / before treatment score × 100%).^[28]^

### 5.3 Exploratory outcome

The exploratory outcomes will be expected to find metabonomics and small molecular marker compounds closely related to the pathogenesis of PMDD. At the same time, combined with the clinical efficacy assessment, the mechanism of Xiaoyaosan in the treatment of PMDD was further explored from the metabolic level, so as to provide a scientific basis for the rational application of this medicine. Urine of the participants will be collected at baseline and at 3 menstrual cycle. Urine will be stored at −20°C until assayed. Non-targeted metabonomics detection of urine will be used to explore the mechanism of PMDD and Xiaoyaosan by liquid chromatograph-mass spectrometer (LC-MS) technology.

## 6 Data collection and management

The data will be collected from record of DRSP and TCM symptom score scale recorded by participants. The scores will be statistically managed by medical officers of the trial team. Data entry and management is the responsibility of the designated data administrator who will establish the symptom database and verification procedures. Two professionally trained operators will enter and confirm the data. After carrying out data logical proofreading, identified errors will be corrected. These data forms will be processed, signed, and dated by the investigator. The database will be locked after the database is established. If any data revision is needed after locking, a research-related signature must be provided.

The Participants are all on the premise of voluntariness in the whole process of the trial. They are allowed to withdraw at any time during the trial. All withdrawn cases will be followed up obtain information about their condition. The data will be analyzed according to the intention-to-treat (ITT) principle.

## 7 Safety assessment

All safety-related variables including vital signs physical examinations, vital signs (temperature, blood pressure, breathing, and heart rate) and laboratory tests (blood count, urine and stool tests, kidney and liver function tests, and electrocardiographic examination) before and after treatment will be recorded. If adverse events (AEs)occur, it will be evaluated by using the National Cancer Institute (Bethesda, MD) Common Terminology Criteria for Adverse Events V.4.03.^[29]^ All AEs will be recorded in the Case Report Forms (CRF). The correlation between adverse reactions and drugs will be evaluated. The researchers decide whether to stop the observation or not according to the situation, and follow-up investigation should be carried out for the cases that are discontinued the observation due to adverse reactions.

## 8 Sample size calculation

This study is a pilot study, so a formal sample size calculation is not completely required.^[30]^ A sample size of 60 participants will be recruited, 30 PMDD participants and 30 healthy participants. Results from this study will be applied to the sample size calculations in future studies.

## 9 Statistical analysis

The data will be analyzed by using Statistical Packages of Social Sciences software (SPSS 24.0). Categorical variables will be described statistically by percentages or frequencies. Chi-square test will be used for comparison between groups. Continuous variables will be described as mean, standard deviation, median, and range. The independent *t* test or Wilcoxon rank-sum test will be used to compare continuous variables. All results presented in this study are based on two-sided tests. The value of *P* < .05 will be considered statistically significant.

## 10 Patient and public involvement

Patients and public were not involved in the design of this study.

## 11 Ethics and dissemination

The trial has been approved by the Institutional Ethics Committee of Dongzhimen Hospital Affiliated to Beijing University of Chinese Medicine (file number: DZMEC-KY-2019–73). The protocol has been registered with Chinese Clinical Trial Registry (identifier ChiCTR1900026296) and conducted in accordance with the Declaration of Helsinki. Written informed consent will be obtained from all participants. Any information of participants will be handled confidentially. The results of the study will be published in peer-reviewed journals or communicated via yearly reports to funding bodies.

## 12 Discussion

PMDD, a severe mental disorder occurring in the luteal phase of women, has a quite negative impact on patient's daily lives. Diagnostically, PMS and PMDD represent the same symptoms,^[31]^ but PMDD is the serious form of PMS.^[32]^ The incidence of PMS is relatively high. About 95% of women of reproductive age have premenstrual symptoms,^[33]^ and 13% to 18% of women need treatment due to PMDD.^[32]^ The common therapy of PMDD is antidepressants. Due to the side effects of drugs for PMDD, such as headache, weight gain, sexual dysfunction, insomnia, and other symptoms, the compliance of patients is rather poor, and then affect the drug efficacy.^[34]^ Therefore, more therapeutic agents than the currently available are needed to improve the quality of these patients life.

Traditional Chinese medicine has a positive effect on improving emotional symptoms and may be the important choice of complementary and alternative medicines for the treatment of PMDD. Chinese herbal medicine treatment is based on the TCM theory of ‘treatment based on syndrome differentiation’. Liver-qi depression provide the crucial pathogenesis of PMDD in the TCM concept. Xiaoyaosan is composed of herbal medicine with the function of regulating emotional factors, which can improve symptoms of depression and anxiety. A Meta-analysis study found that no serious adverse events were reported in ten randomized controlled trials involving Xiaoyaosan intervention.^[35]^ In addition, Compared with antidepressants, Xiaoyaosan plus antidepressants could reduce the adverse reactions.^[35]^ Chinese herbal medicine treatment is based on the TCM theory of ‘treatment based on syndrome differentiation’. Therefore, adding TCM syndrome efficacy evaluation may help to determine more suitable patient populations and optimize indications in this study.

Current clinical studies on PMDD mainly focus on efficacy evaluation, the potential mechanism of drug action has been ignored. So this trail will study the pathogenesis of the PMDD and the action mechanism of TMC from the perspective of metabonomics along the efficacy evaluation of Xiaoyaosan. The metabonomics and small molecular marker compounds closely related to the pathogenesis of PMDD and efficacy mechanism of Xiaoyaosan by the LC-MS technology are expected to be explored. If the research is successful, the results of this trial could provide preliminary objective evidence on the efficacy of Xiaoyaosan for PMDD and more accurate indication of Xiaoyaosan. Significantly, this trial will provide a novel research method for researchers to improve the effectiveness of TCM. However, the limitations of this study include its small sample size, single-center design and short treatment period. Therefore, the larger-sample, large-scale, multi-center, blind, randomized control and longitudinal clinical trials are still needed.

## Acknowledgments

The authors would like to thank Jiuzhitang Co., Ltd., for providing the support of Chinese patent medicine in this study.

## Author contributions

Mengbai Xu, Yanfeng Liu and Jiaxu Chen participated in the design of the study and obtained grant funding. Mengbai Xu drafted the protocol and wrote the original protocol manuscript. Jiaxu Chen and Yu Guo revised the protocol manuscript. Chenyue Liu, Yueyun Liu, Zhiyi Yan, Yajing Hou will collect and analyze the clinical data. Xiaojuan Li, Qingyu Ma, Xuan Zhou, Liuqing Liu, and Sheng Huang will assist in the implementation of the study.

Mengbai Xu orcid: 0000-0001-8211-8800
